# Epidemiological characteristics and low case fatality rate of pandemic (H1N1) 2009 in Japan

**DOI:** 10.1371/currents.RRN1139

**Published:** 2009-12-28

**Authors:** Taro Kamigaki, Hitoshi Oshitani

**Affiliations:** Department of Virology, Tohoku University Graduate School of Medicine

## Abstract

Pandemic (H1N1) 2009 has been causing large outbreaks in Japan. Yet, the case fatality rate (CFR) remains low and only 85 deaths have been confirmed as of December 17, 2009. Surveillance data was analyzed to define epidemiological characteristics of pandemic (H1N1) 2009 in Japan. It was shown that most of the reported influenza-like illness cases and hospitalizations have occurred in those aged 5–9 years and 10–14 years, in whom CFR is extremely low. However, CFRs are higher in small children (<5 years) and adults. The transmission to these age groups may possibly have been minimized through aggressive suspension of classes in schools.

**Introduction**    

Pandemic (H1N1) 2009 (pandemic H1N1) has been spreading worldwide since the first cases were identified in the United States and Mexico in April 2009 [1] [2]The H1N1 virus had caused significant outbreaks in most southern hemisphere countries between May and September 2009. As of early December 2009, it has been spreading in northern hemisphere countries. The case fatality rate (CFR) of pandemic H1N1 was initially estimated to be about 0.4% [3] However, the recent estimate is significantly lower than the initial estimate [4]. Moreover, CFR appears to be different between countries. The World Health Organization (WHO) compared the mortality rates among different countries based on the available data as of early November 2009 [5]. The mortality rates (deaths per million population) ranged from 2.2 to 3.3 in northern hemisphere countries, except in Japan, where the mortality rate was 0.2. In the United States, it is estimated that about 9820 deaths have occurred among 47 million cases [6]. On the other hand, the Ministry of Health, Labour and Welfare (MHLW) of Japan confirmed only 85 deaths as of December 1, 2009, although the estimated number of cases was about 12.6 million by the end of November (week48) [7]. It is widely believed that the low CFR in Japan resulted from aggressive early treatment with antiviral drugs such oseltamivir and zanamivir. In this study, we describe the unique epidemiological characteristics of pandemic H1N1 in Japan, which may be another important factor for low CFR in Japan.    

**Age Distribution of Cases, Hospitalized Cases** **,** **and Deaths in Japan**  

 In Japan, there are about 3000 pediatric and 2000 adult outpatient clinics participating in the influenza sentinel surveillance system that report weekly the number of influenza-like illness (ILI) stratified by age group. Between weeks 28 and 48 of 2009, 1,272,725 ILI cases were reported through the sentinel surveillance system, and it is estimated that 12.6 million people with ILI had visited outpatient clinics [7]. During this period, more than 99% of influenza viruses isolated in Japan were pandemic H1N1. Therefore, it can be assumed that majority of ILI cases during this period were caused by pandemic H1N1. 

**Figure:**
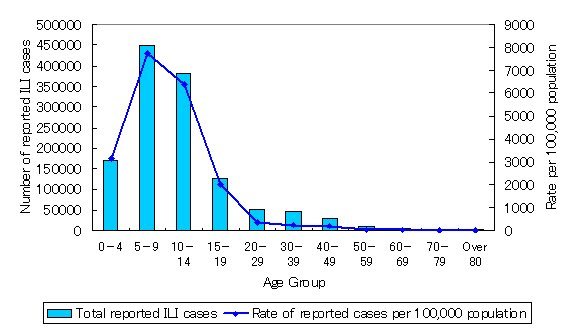

**Figure:**
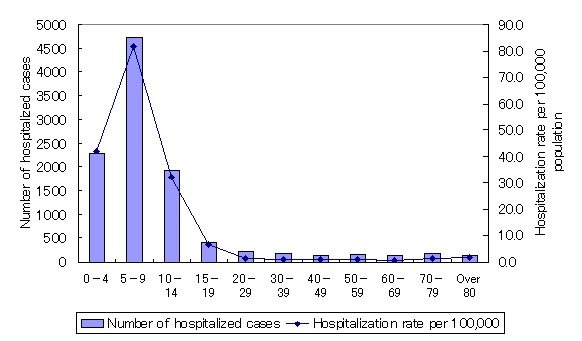

Figure 1. (a) Reported cases of influenza-like illness and rate per 100,000 between week 28 and week 48, 2009 in Japan (Upper). (b) Number of hospitalized cases and hospitalization rate per 100,000 (as of 2 December) (Lower)

Figure 1 shows the age distribution of ILI cases. In all, 1,125,907 ILI cases (88.5%) had occurred in persons younger than 20 years. In other countries, a higher incidence rate was also observed in younger age groups. However, the incidence in older age groups is much lower in Japan than in other countries. Table 1 compares the age distribution patterns of ILI cases between the United States and Japan for pandemic H1N1. Data for the United States was obtained from the Centers for Disease Control and Prevention (CDC) website [8], and Japanese data was obtained from the MHLW website [7]. It is difficult to compare the data directly from ILI surveillance between countries because the systems, including the age groups used for reporting, are different. However, there are some obvious differences between the United States and Japan. First, the proportion of ILI cases in those aged 0–4 years was lower in Japan, and cases in adults were also significantly lower in Japan; more than 75% of cases have occurred in those aged 5–19 years.

**Figure:**
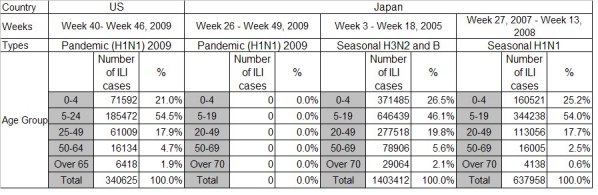

Table 1. Number of reported influenza-like illness (ILI) cases for pandemic (H1N1) in US and Japan and seasonal influenza for 2007-8 and 2004-5 influenza seasons in Japan.  

Table 1 also includes data for the 2004–5 and 2007–8 influenza seasons in Japan. The predominant strains were H3N1 and influenza B for the 2004–5 season and seasonal H1N1 for the 2007–8 season. In both seasons, the proportions of small children (<5 years old) and adults (≥20 years old) with ILI were higher than those for pandemic H1N1 in Japan. Actually, the age distribution patterns in these seasons are more similar to those for pandemic H1N1 in the United States.

Enhanced surveillance of hospitalized cases of pandemic H1N1 has been implemented in Japan since July 2009. Testing with a real-time polymerase chain reaction (PCR) is being conducted for all hospitalized cases with suspected pandemic H1N1 to confirm infection; positive cases are reported to the MHLW. As of December 2, 2009, 10,487 hospitalized cases have been reported to the MHLW [9]. Figure 2 shows the number of hospitalized cases and the hospitalization rate per 100,000 population by age groups. Again, the number of hospitalized cases was highest among those aged 5–9 years, and very low hospitalization rates were observed among adult age groups. In all, 4725 hospitalized cases (45.1%) have occurred in children aged 5–9 years, and 1929 (18.4%) in those aged 10–14 years. In New South Wales, Australia, 1214 hospitalized cases had been identified by August 31, 2009 [10]. Only 69 (5.7%) patients were aged 5–9 years, and 44 (3.6%) were aged 10–14 years. The data of hospitalized cases in the United States between April and June 2009 also indicated that only 11% of total hospitalized cases were aged 5–9 years [11].

**Figure:**
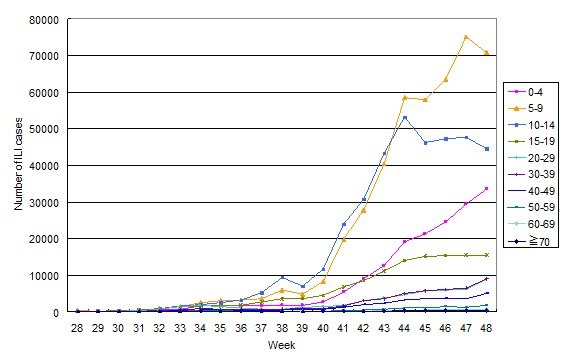

Figure 2. Number of influenza-like illness (ILI) cases reported from sentinel sites in Japan from week 28 to week 48, 2009

**Table:** 

** Age Group **	** Number of Deaths **	** Percentage (%)**	** Mortality rate per 1 million population **	** Mortality rate per estimated 100,000 cases **	** Mortality rate per 100 hospitalizations **
** 0-4**	11	12.9	2.04	0.92	0.48
** 5-9**	8	9.4	1.38	0.24	0.17
** 10-14**	4	4.7	0.67	0.11	0.21
** 15-19**	1	1.2	0.16	0.05	0.24
** 20-29**	4	4.7	0.27	0.39	1.90
** 30-39**	8	9.4	0.43	1.05	4.60
** 40-49**	10	11.8	0.62	1.98	6.94
** 50-59**	10	11.8	0.57	5.27	6.17
** 60-69**	9	10.6	0.53	11.87	6.34
** Over 70 **	20	23.5	0.99	31.65	6.35
** Total**	85	100.0	0.67	0.67	0.81

Table 2. Number of deaths due to pandemic (H1N1) and mortality rates per 1 million population, 100,000 estimated influenza-like illness cases (i.e. case fatality rate) and 100 hospitalizations in Japan.   

As of December 1, 85 deaths had been confirmed in Japan [9]. Among 85 fatal cases, 28 (32.9%) were younger than 20 years (Table 2). In other countries, the proportion of deaths in children is much smaller. In New South Wales, Australia, only one child death occurred among 48 deaths, and the other 47 deaths occurred in those ≥20 years [10]. In California, among 118 deaths, only 8 (6.8%) were younger than 18 years [12]. The mortality rates per 1 million population, per 100,000 estimated cases, and per 100 reported hospitalized cases are also shown in Table 2. The mortality rate per 1 million population was highest in the 0–4-year group, followed by the 5–9-year group and over 70 years. On the other hand it was lowest in the 15–19-year group. The mortality rates per estimated cases and per reported hospitalizations were very low among age groups of 5–9 years, 10–14 years, and 15–19 years. These mortality rates were higher in children aged 0–4 years than in older children. Moreover, the mortality rates, both per estimated cases and per reported hospitalizations, increased significantly with age in adults. The mortality rate per estimated cases in the 50–59-year group was more than 100 times higher than that in the 15–19-year group. The mortality rates per hospitalization were also much higher in adults, particularly in those ≥40 years. Our data indicate that CFR in small children (i.e., <5 years) and adults, especially the elderly, are higher than among those aged 5–19 years. However, most of the infections to date have occurred in these age groups (i.e., 5–19 years), while infections in other age groups are still very limited. This may be one possible reason why the CFR in Japan remains low.  

**School closures and epidemiological characteristics**

In the previous section, we showed that the epidemiological characteristics of pandemic H1N1 are unique in Japan, which may have kept the CFR low. The main question arises: why are there unique epidemiological characteristics in Japan? The majority of cases have occurred in age groups of 5–9 years and 10–14 years (Figure 1). In general, children in primary school are aged between 6 and 12 years, and those in junior high school are aged between 13 and 15 years. Therefore, the current age distribution of cases indicates that the majority of pandemic H1N1 cases in Japan have occurred among children in primary and junior high schools. Both pandemic and seasonal influenza outbreaks often start as school outbreaks, which often become a trigger for community outbreaks. This is why early school closures or suspension of classes can be effective in reducing transmission into the community [13]. In Japan, suspension of classes is commonly implemented even for seasonal influenza [14]. For example, during the 2006–7 influenza season, 14,103 institutions (including day care centers, kindergartens and primary, junior high, and high schools) suspended classes [15]. An even more aggressive suspension of class policy has been implemented for pandemic H1N1 in 2009. Between October 25 and December 5, 2009, 94,781 institutions had implemented suspension of classes [16]. On the other hand, the CDC of the United States is not recommending such aggressive measures in the school setting [17].

Figure 2 shows the numbers of reported ILI cases by age groups in Japan. The numbers of cases among children aged 5–9 years and 10–14 years increased sharply after week 40. The highest number of cases occurred in week 44 for children aged 10–14 years and in week 47 for those aged 5–9 years, which had the highest peak on week 48. On the other hand, the number of cases in children aged 0–4 years has been increasing, particularly after week 43, and the number in adult age groups has been increasing even after week 47. These findings suggest that outbreaks in primary and junior high schools had already reached the peak by week 47, but until about week 42, the transmission into households may have been minimized by suspending classes early. However, it appears that the transmission occurring outside schools, including within households, are increasing. We have proposed the concept that with progression of outbreaks of pandemic H1N1 the incidence frequency of the outbreak shifts from schools to community, which may increase the occurrence of more severe cases and deaths resulting in higher CFR [18].  

**Discussion**  

In Japan, despite a widespread transmission of pandemic H1N1, the CFR is still low. It is widely believed that the low CFR in Japan resulted from early treatment with antivirals. In fact, Japanese physicians frequently prescribe neuraminidase inhibitors (oseltamivir or zanamivir) even for seasonal influenza. The majority of pandemic H1N1 cases are likely to have received neuraminidase inhibitors in Japan. The MHLW published the data on severe cases on November 20, 2009 [19]. They analyzed 50 fatal cases in Japan and found that 26% of fatal cases received antiviral treatment on the day of onset and 30% on the day following onset of illness. This data suggests that even early treatment with antivirals cannot prevent a fatal outcome in some cases. There is also a recent debate that questions the effectiveness of neuraminidase inhibitors in reducing complications for seasonal influenza [20],[21]. Currently available evidence on effectiveness of neuraminidase inhibitors to reduce severity of pandemic H1N1 is also not very strong, and all data are based on observational findings [22]. For example, it has been shown that hospitalized cases who received early treatment were less likely to require intensive care or were less likely to die [12]. However, those who received early treatment may have had a different demographic background, or may also have received other supportive care, such as oxygen therapy, early enough to prevent severe complications. Further studies are needed to define the effect of neuraminidase inhibitors in reducing the severity of pandemic H1N1. At present, it is not possible to conclude that neuraminidase inhibitors are effective in reducing severe infection of pandemic H1N1 solely because of the low CFR in Japan, where neuraminidase inhibitors are used extensively.

In this study we have discussed another possible reason for the low CFR in Japan. Epidemiological characteristics of pandemic H1N1 in Japan are, to date, unique. Most cases and hospitalizations have occurred in those aged between 5 and 14 years. Children in these age groups are highly susceptible to infection of pandemic H1N1, but they are less likely to develop severe complications, and the CFR is extremely low. On the other hand, severe complications are more common in smaller children aged <5 years and adults aged ≥30 years, particularly in the elderly. However, the attack rate in these age groups is relatively low in Japan. It is still not clear what has produced this epidemiological pattern in Japan. The measure to aggressively suspend classes in schools may have contributed to a reduction in transmission into the community. However, cases in smaller children and adults are gradually increasing. More recent data indicated that the number of deaths particularly in adult patients was increasing. By December 17, 2009, according to the MHLW, 122 deaths have been confirmed in Japan. Among these cases, 37 died in December, 29 (78%) of whom were older than 40 years. This data suggests an increasing trend toward fatal cases in the adult population. The overall mortality impact in Japan may depend on how infections are spreading in different age groups in coming months.   

**Funding Information**

Funding was provided in part by research project for emerging and re-emerging infectious diseases, Ministry of Health, Labour and Welfare, Japan.

**Competing Interests**

All of the authors declare that no competing interests exist.
